# Local apoptosis promotes collagen production by monocyte-derived cells in transforming growth factor β1-induced lung fibrosis

**DOI:** 10.1186/1755-1536-4-12

**Published:** 2011-05-17

**Authors:** Xueyan Peng, Susan K Mathai, Lynne A Murray, Thomas Russell, Ronald Reilkoff, Qingsheng Chen, Mridu Gulati, Jack A Elias, Richard Bucala, Ye Gan, Erica L Herzog

**Affiliations:** 1 Department of Internal Medicine, Yale University School of Medicine, 300 Cedar Street, TAC 441S, New Haven, CT, USA; 2 Medimmune Ltd, Granta Park, Cambridge, CB21 6GH, UK; 3 Department of Medicine, Central South University, Changsha, Hunan China

## Abstract

**Background:**

Collagen-containing leukocytes (CD45^+^Col-I^+^) accumulate in diseased and fibrotic tissues. However, the precise identity of these cells and whether injury is required for their recruitment remain unknown. Using a murine model of pulmonary fibrosis in which an inducible, bioactive form of the human transforming growth factor (TGF)-β1 gene is targeted to the lung, we characterized the cell surface phenotype of collagen-containing CD45^+ ^cells in the lung and tested the hypothesis that apoptotic cell death responses are essential to the accumulation of CD45^+^Col-I^+ ^cells.

**Results:**

Our studies demonstrate that CD45^+^Col-I^+ ^cells appearing in the TGF-β1-exposed murine lung express markers of the monocyte lineage. Inhibition of apoptosis via pharmacological caspase blockade led to a significant reduction in CD45^+^Col-I^+ ^cells, which appear to accumulate independently of alternatively activated macrophages. There are also increased levels of apoptosis and greater numbers of CD45^+^Col-I^+ ^in the lung tissue of patients with two distinct forms of fibrotic lung disease, idiopathic pulmonary fibrosis and connective tissue disease-related interstitial lung disease, when compared to lung from healthy normal controls. These findings are accompanied by an increase in collagen production in cultured monocytes obtained from subjects with fibrotic lung disease. Treatment of these cultured cells with the caspase inhibitor carbobenzoxy-valyl-alanyl-aspartyl-[*O*-methyl]-fluoromethylketone (Z-VAD/fmk) reduces both apoptosis and collagen production in all subjects.

**Conclusions:**

Interventions that prevent collagen production by monocytes via modulation of caspase activation and of apoptosis may be ameliorative in monocyte-associated, TGF-β1-driven processes such as pulmonary fibrosis.

## Background

The CD14^+ ^fraction of peripheral blood contains heterogeneous monocyte progenitors with important roles in tissue injury and repair [1]. A subpopulation of monocytes differentiates into fibrocytes by acquiring a fibroblast-like morphology, gaining expression of collagen I and CD34 while losing CD14 expression [2]. Fibrocytes accumulate in transforming growth factor (TGF)-β1-exposed tissues [3] and are associated with an array of fibrosing disorders including asthma, pulmonary fibrosis, and scleroderma [4-6]. Due to the considerable variability in methods used to identify these cells, controversy exists as to their true phenotype [7,8] though the presence of fibrocytes in several forms of fibrosis is now well established. The mechanism(s) through which fibrocytes and related CD45^+ ^collagen (Col)-I^+ ^cells contribute to fibrosis remain unclear, but may be related to immunological regulation of effector cell phenotypes [9] as well as direct production of extracellular matrix proteins or α-smooth muscle actin (SMA) production [10,11]. This phenotype is specialized for the characteristics that might be required for repair. However, while the administration of human fibrocytes to severe combined immunodeficiency (SCID) mice requires coadministration of bleomycin to result in pathology [12], requirement for injury in the accumulation of CD45^+^Col-I^+ ^in the TGF-β1-exposed murine lung has not been shown.

Pulmonary fibrosis is a progressive and often fatal disease for which there are no effective therapies. The current paradigm of pulmonary fibrosis pathogenesis includes recurrent epithelial cell death responses with subsequent recruitment of a monocyte-derived inflammatory infiltrate and the eventual development of myofibroblast activation [13]. These events are believed to be heavily influenced by TGF-β1 [14-17]. While the precise type of injury initiating these events remains unknown, substantial evidence supports a role for apoptosis as a contributing factor [18-20]. Elevations in circulating and/or tissue CD45^+^Col-I^+ ^cells have are seen in a broad array of fibrosing lung diseases including idiopathic pulmonary fibrosis (IPF) [4], asthma [5], post-transplant bronchiolitis obliterans syndrome [21], and scleroderma [6]. Many of these diseases are associated with abnormalities in apoptosis [19,22,23]; however, a relationship between CD45^+^Col-I^+ ^cells, specifically fibrocytes, and apoptosis has not been previously assessed.

We have recently shown that transgenic overexpression of TGF-β1 results in the accumulation of cells that coexpress CD45 and Col-Iα1 [24]. However, the cell surface phenotype of these cells remains unexplored and the local events initiating TGF-β1-induced accumulation of CD45^+^Col-Iα1^+ ^cells remain obscure. Because the TGF-β1 phenotype requires apoptosis for the development of fibrosis and remodeling [18] we thought it likely that the increase in CD45^+^Col-Iα1^+ ^cells seen in this model were caused by increases in this form of cell death. To test this hypothesis we explored the identity of CD45^+^Col-Iα1^+ ^cells in a mouse model of pulmonary fibrosis caused by transgenic overexpression of the bioactive human TGF-β1 gene and examined whether caspase-mediated apoptotic responses are required for the appearance of these cells. The human relevance of these findings was explored in studies of cultured cells obtained from patients with multiple forms of pulmonary fibrosis. Our results indicate that CD45^+^Col-Iα1^+ ^cells recruited to the lung by TGF-β1 are enriched for the expression of CD14 and that their appearance in the lung requires an increase in apoptotic cell death responses. Our data also demonstrate that CD14^+ ^monocytes derived from the circulation of patients with multiple forms of lung fibrosis show robust CD34 expression and display a propensity for collagen production that is reduced when apoptosis is blocked.

## Results

### Collagen-containing leukocytes are a heterogeneous cell population

We have previously shown that inducible overexpression of the human TGF-β1 gene results in the accumulation of CD45^+^Col-Iα1^+ ^cells in the murine lung [3,25]. While this combination of markers has traditionally been considered sufficient for the identification of fibrocytes [8], accumulating data from our group and from others indicate that this combination of markers may in fact identify a heterogeneous population of cells [6,7]. Thus, in order to better characterize the identity of TGF-β1-recruited intrapulmonary CD45^+^Col-Iα1^+ ^cells, TGF-β1 transgenic positive (Tg^+^) and wild-type control (Tg^-^) mice received doxycycline in their drinking water for up to 2 weeks after which they were killed and CD45^+^Col-Iα1^+ ^cells quantified as we have previously described [3]. While we would have preferred to use an antibody specific for the immature form of collagen I, such an antibody is currently not available. Thus, detection of the mature form of collagen was employed. These cells were then further immunophenotyped based on their expression of CD14 and/or CD34.

**Figure 1:**
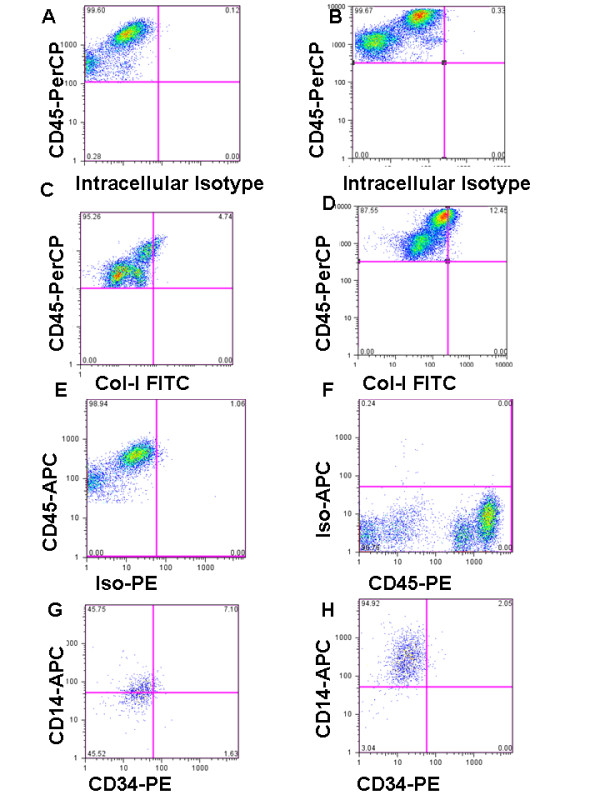
**Flow cytometric detection of collagen containing monocytes in the transforming growth factor (TGF)-β1-exposed murine lung**. **(a,b) **Fluorescein isothiocyanate (FITC)-detected intracellular isotype control (x axis) versus CD45-peridinin chlorophyll protein complex (PerCP) (y axis) in (a) Tg^- ^and (b) Tg^+ ^mice. This control was used to set the negative gate for FITC in each animal respectively. A shift in autofluorescence is seen in the Tg^+ ^animals. **(c,d) **FITC-detected collagen (Col)-Iα1 (x axis) versus CD45-PerCP (y axis) in (c) Tg- and (d) Tg^+ ^mice. An increase in double positive cells is seen in the Tg^+ ^animals. **(e) **Phycoerythrin (PE) -isotype control (x axis) versus CD45-anti-allophycocyanin (APC) (y axis). This control was used to set the negative gate for PE. **(f) **CD45-PE (x axis) versus APC-isotype control (y axis). This control was used to set the negative gate for APC. **(g,h) **CD34 (x axis) and CD14 (y axis) on the CD45^+^Col-Iα1^+ ^population identified in (g) Tg^- ^and (h) Tg^+ ^mice. In contrast to Tg- mice, in which the CD45^+^Col-Iα1^+ ^cells express CD14 and CD34 to varying degrees, the CD45^+^Col-Iα1^+ ^cells that appear in response to TGF-β1 overexpression are almost uniformly CD14^+^, with minimal contribution of CD34^+ ^cells. N = 5 mice/group, images are representative of all animals.

**Figure 2:**
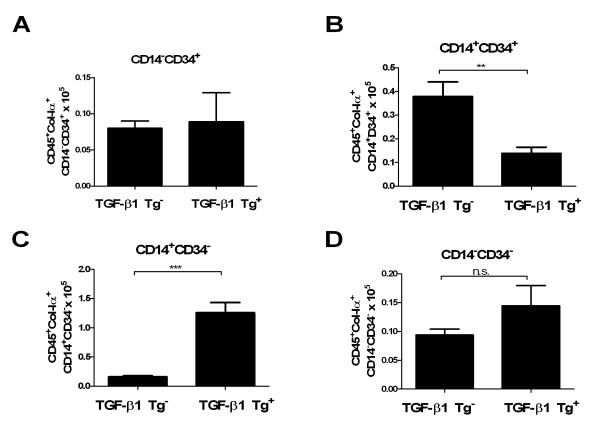
**Expression of cell surface markers on CD45^+ ^collagen (Col)-Iα1^+ ^cells from transforming growth factor (TGF)-β1-exposed murine lung**. **(a) **Quantities of CD45^+^Col-Iα1^+^CD14^-^CD34^+ ^cells did not differ between the TGF-β1 Tg^- ^and Tg^+ ^mice. **(b) **CD45^+^Col-Iα1^+^CD14^+^CD34^+ ^cells were significantly reduced in the TGF-β1 Tg^+ ^mice. **(c) **CD45^+^Col-Iα1^+^CD14^+^CD34^- ^cells were significantly increased in the TGF-β1 Tg^+ ^mice. **(d) **CD45^+^Col-Iα1^+^CD14^-^CD34^-^cells did not differ between TGF-β1 Tg^- ^and TGF-β1 Tg^+ ^mice. N = 5 mice/group, images are representative of all animals. **P < 0.01, ***P < 0.001.

Consistent with our prior findings, CD45^+^Col-Iα1^+ ^cells were detected in all mice, with a robust increase seen in the TGF-β1 Tg^+ ^animals (Figure 1a-d). Further assessment revealed that in all mice these cells displayed variable expression of CD14 and CD34 (Figure 1e-h). Interestingly, cells meeting classical definition of fibrocytes based upon the coexpression of CD34, CD45, and Col-Iα1 in the absence of CD14, were rare in both sets of animals and not significantly altered between groups (Figure 2a). In contrast, when compared to Tg^- ^animals, the lungs of TGF-β1 Tg^+ ^mice contained 64.8% fewer CD45^+^Col-Iα1^+ ^CD14^+^CD34+^+ ^cells (*P *< 0.001, Figure 2b) but nearly tenfold more CD45^+^Col-Iα1^+ ^CD14^+^CD34^- ^cells (*P *< 0.001, Figure 2c). The amount of CD45^+^Col-Iα1^+ ^cells expressing neither CD14 nor CD34 (CD45^+^Col-Iα1^+ ^CD14^-^CD34^-^) did not differ between groups (Figure 2d). These data indicate that CD45^+^Col-Iα1^+ ^+ cells appearing in the TGF-β1-exposed lung are primarily composed of cells that express CD14 and lack CD34.

### Caspase inhibition attenuates TGF-β1-induced apoptosis and accumulation of CD45^+^Col-Iα1^+ ^cells

**Figure 3:**
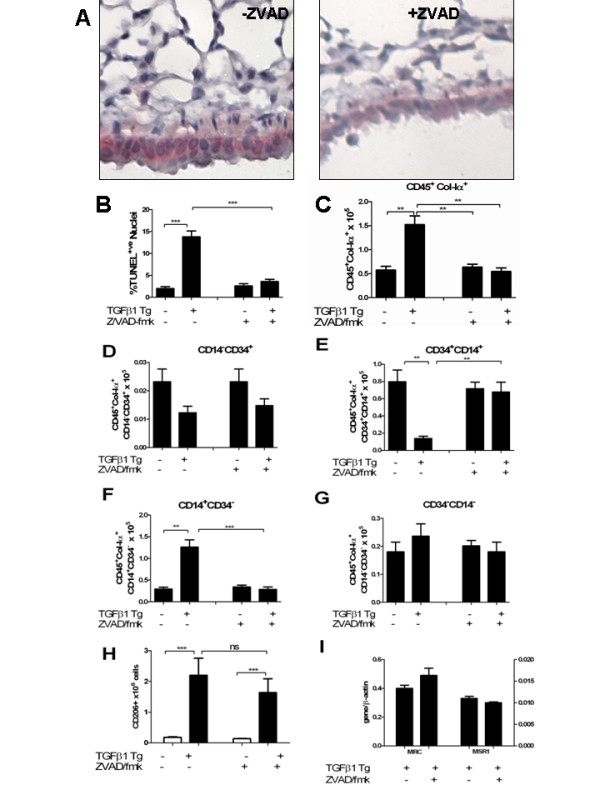
**Effect of caspase inhibition of transforming growth factor (TGF)-β1-induced apoptosis and CD45^+ ^collagen (Col)-Iα1^+ ^cell accumulation**. **(a) **Cleaved caspase-3 immunohistochemistry (red) in TGF-β1 Tg**^+ ^**mice exposed to vehicle (left) or carbobenzoxy-valyl-alanyl-aspartyl-[*O*-methyl]-fluoromethylketone (Z-VAD/fmk) (right). Nuclei are counterstained with hematoxylin, 40 × original magnification. A significant reduction in cleaved (activated) caspase-3 is noted in the Z-VAD/fmk treated mice. **(b,c) **Z-VAD/fmk reduces terminal deoxynucleotidyl transferase dUTP nick end labeling (TUNEL) staining (b) and CD45^+^Col-Iα1^+ ^cells (c) in TGF-β1 Tg^+ ^mice. **(d-f) **Compared to untreated mice, the lungs of TGF-β1 Tg^+ ^treated with Z-VAD/ fmk demonstrate unchanged quantities of CD45^+^Col-Iα1^+^CD14^-^CD34^+ ^cells (d), increased quantities of CD45^+^Col-Iα1^+^CD14^+^CD34^+^cells (e), reduced quantities of CD45^+^Col-Iα1^+^CD14^+^CD34^- ^cells, and unchanged quantities of CD45^+^Col-Iα1^+^CD14^-^CD34^- ^cells. **(h) **Accumulation of CD206/MRC^+ ^cells is not affected by Z-VAD/fmk. **(i) **TGF-β1-induced expression of the scavenger receptors MRC (left axis) and MSR-1 (right axis) is not affected by administration of Z-VAD/fmk. N = 5 mice/group. *P < 0.05, **P < 0.01, ***P < 0.001.

In order to explore the role of intrapulmonary caspase activation and apoptotic responses in the accumulation and phenotype of CD45^+^Col-Iα1^+^cells, TGF-β1 Tg^+ ^and Tg^-- ^mice were given doxycycline in their drinking water and randomized to receive intraperitoneal dosing of the caspase inhibitor carbobenzoxy-valyl-alanyl-aspartyl-[*O*-methyl]-fluoromethylketone (Z-VAD/fmk) for between 2 to 14 days. Mice were killed at the height of cell death, which occurs at 48 hours in the model, and assessed for caspase-3 activation using immunohistochemistry and for cell death responses using terminal deoxynucleotidyl transferase dUTP nick end labeling (TUNEL) staining. Consistent with our prior reports [18], caspase-3 activation was abrogated in the presence of Z-VAD/fmk (Figure 3a) and assessment of TUNEL staining in the TGF-β1 Tg^+ ^lung revealed a 79.9% reduction in cell death responses at this timepoint (*P *< 0.0003, Figure 3b).

Having confirmed that caspase inhibition does indeed reduce apoptosis in this model, we next explored its effects on the recruitment of CD45^+^Col-Iα1^+ ^cells. Here we found that treatment of TGF-β1 Tg^+ ^mice with Z-VAD/fmk reduced CD45^+^Col-Iα1^+ ^cells by nearly tenfold (*P *< 0.0014, Figure 3c) and restored quantities of all CD45^+^Col-Iα1^+ ^subtypes to wild type levels. Specifically, compared to sham-treated TGF-β1 Tg^+ ^mice, the lungs of Z/VAD-fmk treated Tg+ mice showed no change in CD45^+^Col-Iα1^+^CD14^-^CD34^+ ^cells (*P *= 0.14, Figure 3d), an 85.8% reduction in CD45^+^Col-Iα1^+^CD14^+^CD34^- ^cells (*P *< 0.001, Figure 3e), a 71.8% increase in CD45^+^Col-Iα1^+^CD14^+^CD34^+ ^cells (*P *< 0.001, Figure 3f), and essentially no change in CD45^+^Col-Iα1^+^CD14^-^CD34^- ^cells (*P *= 0.35, Figure 3g). These data indicate that apoptotic cell death responses regulate the appearance and phenotype of CD45^+^Col-Iα1^+ ^cells in the TGF-β1-exposed murine lung.

### Collagen-producing leucocytes accumulate independently of alternatively activated macrophages

Our prior studies have revealed that alternatively activated (M2) macrophages regulate the development of fibrosis [3,26]. However, the precise relationship between fibrocytes and macrophages in the TGF-β1-exposed lung has not been fully explored. Given the importance of the M2 macrophage in tissue repair and remodeling responses we thought it possible that M2 macrophages control the appearance of CD45^+^Col-Iα1^+ ^cells in our model. In order to test this hypothesis, the effect of caspase inhibition on CD206/MRC^+ ^alternatively activated macrophages was assessed via flow cytometry as we have previously described [3]. Results of these studies revealed only a trend toward reduced M2 macrophages in the Z/VAD-fmk treated mice that did not reach statistical significance (*P *= 0.09, Figure 3h). Analysis of M2-related genes such as CD206/MRC and MSR-1 using quantitative RT-PCR confirmed these results (*P *> 0.05 both comparisons, Figure 3i). Because caspase inhibition caused a profound reduction in CD45^+^Col-Iα1^+ ^cells without a concomitant reduction in M2 macrophages, the expression of collagen by monocyte-derived cells is unlikely to be controlled solely by accumulation of M2 macrophages.

### Intrapulmonary apoptosis and CD45^+^Pro-Col-Iα1^+ ^cells are increased in patients with lung fibrosis

**Figure 4:**
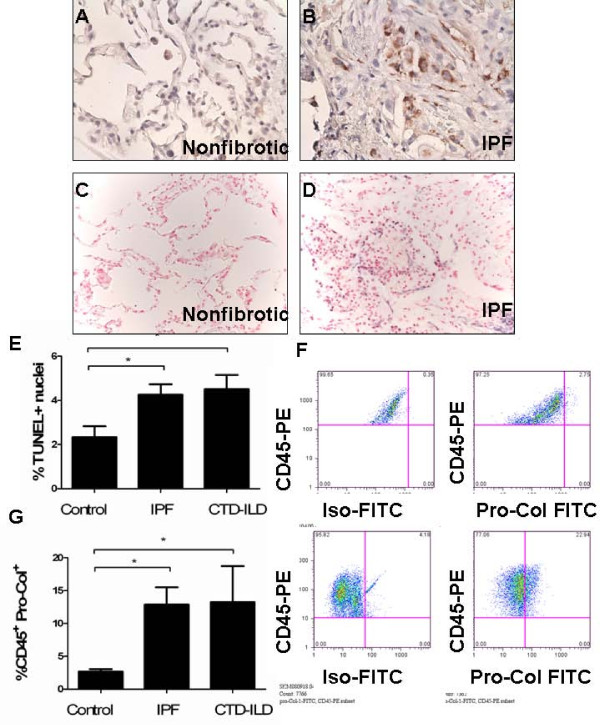
**Fibrotic human lungs are enriched for terminal deoxynucleotidyl transferase dUTP nick end labeling (TUNEL) staining and CD45^+^Pro-collagen (Col)-Iα1^+ ^cells**. **(a,b) **Assessment of cleaved caspase 3 (brown cytoplasmic stain) on lung biopsy sample from (a) non-fibrotic and (b) idiopathic pulmonary fibrosis (IPF) lung sample. Nuclei are counterstained with hematoxylin (60 × original magnification). An increase in caspase cleavage is seen in the IPF lung. **(c,d) **TUNEL staining (black nuclear stain) performed on (c) non-fibrotic and (d) IPF lung shows an increase in TUNEL+ nuclei in the IPF lung. Nuclei are counterstained with nuclear fast red, 40 × original magnification. **(e) **Compared to non-fibrotic samples (left) TUNEL staining is increased in lungs obtained from subjects with IPF (middle) or connective tissue disease interstitial lung disease (CTD-ILD) (right). **(f) **CD45^+^Pro-Col-Iα1^+ ^cells are increased in fibrotic lungs. Top left: intracellular isotype (x axis) versus CD45-PE (y axis) on cells obtained from a non-fibrotic lung. The diagonal morphology of the cells reflects the high inherent autofluorescence of lung inflammatory cells. Top right: Pro-Col-Iα1- fluorescein isothiocyanate (FITC) (x axis) versus CD45-PE (y axis) on non-fibrotic lung. There is only minimal shift past the isotype control. Bottom left: intracellular isotype (x axis) versus CD45-PE (y axis) in cells obtained from an IPF lung. Bottom right: Pro-Col-Iα1-FITC (x axis) versus D45-PE (y axis) on IPF lung. **(g) **Compared to non-fibrotic subjects (n = 6) (left), detection of CD45^+^Pro-Col-Iα1^+ ^cells is increased in the lungs of subjects with either IPF (n = 4, middle) or CTD-ILD (n = 4, right). *P < 0.05 **P < 0.01.

We next sought to determine the human relevance of these findings. In planning these studies we reasoned that if collagen-production in monocytes demonstrated a biological relationship with the development of fibrotic lung disease, then they would be detected in multiple forms of lung fibrosis. Thus, our murine studies were recapitulated in lung tissue from the discarded surgical margins of biopsy samples from patients with histopathological or clinical findings consistent with IPF or clinical diagnosis of connective tissue disease interstitial lung disease (CTD-ILD), or subjects with no known parenchymal lung disease. Immunohistochemistry performed on these samples revealed increased caspase 3 cleavage in the fibrotic samples (Figure 4a, b and data not shown) and a nearly twofold increase in TUNEL staining in both the IPF samples and CTD-ILD samples when compared to non-fibrotic control (*P *< 0.05 both comparisons, Figure 4c-e). Furthermore, while non-fibrotic lungs contained relatively low numbers of CD45^+^Pro-Col-Iα1^+ ^cells, quantities of this population were increased nearly threefold in the samples with IPF and CTD-ILD (*P *< 0.05 for both comparisons, Figure 4e). Notably, accumulation of intrapulmonary CD45^+^Pro-Col-Iα1^+ ^cells did not differ between IPF and CTD-ILD groups (Figure 4f). These data indicate that the lungs of patients with various forms of lung fibrosis demonstrate increased apoptosis and elevated numbers of CD45^+^Pro-Col-Iα1^+ ^cells.

### Cultured monocytes from humans with lung fibrosis display enhanced differentiation into fibrocytes

**Figure 5:**
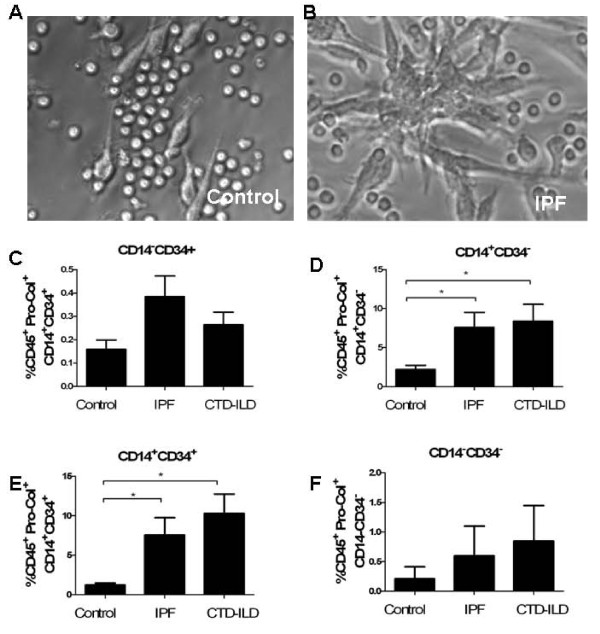
**Cultured CD45 ^+ ^Pro-collagen (Col)-Iα^+ ^cells obtained from patients with pulmonary fibrosis demonstrate significant heterogeneity**. **(a,b) **Spindle-shaped cells in cultured monocytes obtained from (a) control samples and (b) idiopathic pulmonary fibrosis (IPF) samples. The increase in number of spindle-shaped cells is seen in suggests an increase in fibrocytes. Similar results were seen in connective tissue disease interstitial lung disease (CTD-ILD) (data not shown). **(c-f) **Immunophenotyping of the cultured cells shown in (a) and (b). **(c) **In contrast to CD45^+^Pro-Col-Iα1^+^CD34^+^CD14^- ^cells, which are quite rare and do not differ in frequency between normal (n = 8), (c), IPF (n = 7), and CTD-ILD (n = 11), CD45^+^Pro-Col-Iα1^+^CD34^-^CD14^+ ^cells (d), as well as CD45^+^Pro-Col-Iα1^+^CD34^+^CD14^+ ^cells (e) are increased in IPF and CTD-ILD patients. CD45^+^Pro-Col-Iα1^+^CD34^-^CD14^- ^cells did not differ between groups (f). Among the patients with CTD-ILD, subgroup analysis did not detect a difference between systemic sclerosis (SSc)-ILD and amyopathic antisynthetase syndrome (AMAS) patients (data not shown). *P < 0.05 **P < 0.01 ***P < 0.001.

The pathogenesis of pulmonary fibrosis is thought to result in part from repeated bouts of injury leading to ongoing remodeling responses and dysregulated repair. The finding that fibrotic lung tissue is enriched for both apoptotic cell death responses and increased quantities of CD45^+^Pro-Col-Iα1^+ ^cells supports an association between these processes. A mechanistic connection was explored in a human lab sample routinely accessible in clinical medicine: the peripheral blood. Here, circulating monocytes were obtained from the peripheral blood of patients with IPF and CTD-ILD, as well as of normal healthy controls, and cultured under serum-containing conditions that favor fibrocyte outgrowth [27]. Characteristics of subjects are shown in Table 1. Assessment of spindle-shaped cells, which have traditionally been considered to be fibrocytes [2,7], revealed increased outgrowth of fibrocytes in the patients with ILD (Figure 5a, b and data not shown). Assessment of collagen expression via flow cytometry revealed that collagen expression was augmented in the subjects with IPF and CTD-ILD as well. Further analysis of phenotype revealed that total percentages of CD45^+^Pro-Col-Iα1^+^CD14^-^CD34^+ ^cells were quite low in cultures from all groups (*P *= 0.09, Figure 5c). In contrast, CD45^+^Pro-Col-Iα1^+^CD14^+^CD34^- ^cells were low in healthy subjects but increased by threefold to fourfold in the IPF and CTD-ILD samples (*P *< 0.02, Figure 5d). Percentages of CD45^+^Pro-Col-Iα1^+^CD14^+^CD34^+ ^cells were low in controls but even further increased in the IPF and CTD-ILD subjects (*P *< 0.02, Figure 5e). Cells exhibiting expression of neither marker (CD45^+^Pro-Col-Iα1^+^CD14^-^CD34^-^) were rare in all subjects (*P *= 0.65, Figure 5f). Subgroup analysis of the CTD-ILD samples did not reveal a difference between disease subtypes (data not shown).

**Table 1:** Characteristics of subjects

	UIP/IPF, N = 7	CTD-ILD, N = 11	Controls, N = 8	*P *value
Age, years	74.14 ± 2.132	55.73 ± 4.673	64.25 ± 18.0	NS

Sex, female	3/7	6/11	6/8	NS

Race:				

Caucasian	7	7	6	

African-American	0	2	0	

Asian	0	2	2	

Disease subtype:				

SSc-ILD (NSIP pattern based on CT scan)	N/A	6/11	N/A	

AMAS-UIP pattern (biopsy proven)		5/11		

Known pulmonary hypertension	0/9	0/11	N/A	N/A

FVC, percentage of predicted	70.86 ± 2.492	60.70 ± 4.410	N/A	NS

DLCO, percentage of predicted	55.29 ± 5.643	56.82 ± 5.220	N/A	N/A

AMAS = amyopathic antisynthetase syndrome; CT = computed tomography; CTD = connective tissue disease; DLCO = diffusing capacity of the lung for carbon monoxide; FVC = forced vital capacity; IPF = idiopathic pulmonary fibrosis; ILD = interstitial lung disease; N/A = not applicable; NS = not significant; NSIP = non-specific interstitial pneumonia; SSc = systemic sclerosis; UIP = usual interstitial pneumonia.

### Caspase inhibition attenuates collagen production in cultured monocytes

**Figure 6:**
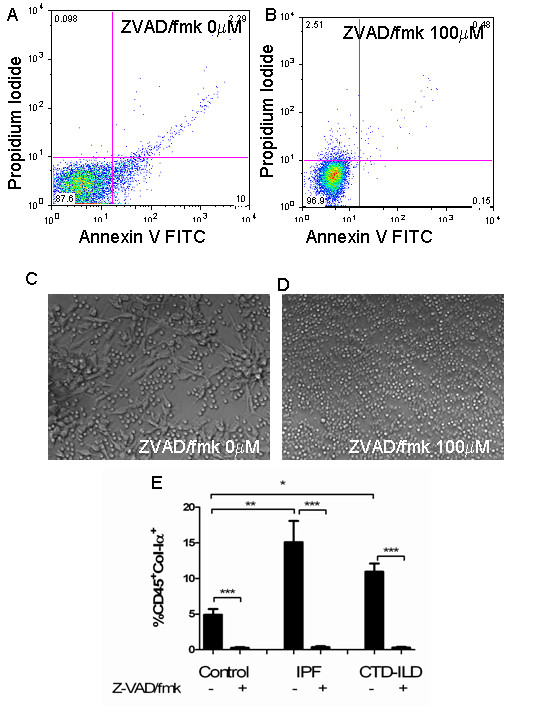
**Caspase inhibition administration attenuates apoptosis and collagen production in cultured human monocytes**. **(a,b) **Flow cytometric quantification of apoptosis based on annexin V (x axis) and propidium iodide (y axis) in cultured idiopathic pulmonary fibrosis (IPF) monocytes reveals that annexin V^+ ^cells are reduced in the setting of Z-VAD/fmk. **(c,d) **Assessment of cultured connective tissue disease interstitial lung disease (CTD-ILD) monocytes indicates that, compared to mock-treated cells, cultured monocytes fail to adopt a spindle-shaped phenotype when treated with Z/VAD-fmk. Similar results are seen in patients with IPF (data not shown) **(e) **Treatment with Z-VAD/fmk attenuates collagen production in samples from all groups. *P < 0.05, **P < 0.01, ***P < 0.001.

Finally, we determined whether caspase inhibition affected the phenotype of cultured monocytes from human subjects in the three groups. Cultured monocytes from each group were treated with 100 mM of Z-VAD/fmk or phosphate-buffered saline (PBS) control and assessed for changes in apoptosis and collagen production. Quantification of cellular apoptosis using annexin V labeling indicated a near complete eradication of apoptosis in the Z-VAD/fmk-treated cells (Figure 6a, b). These cells included cells in the early stages of apoptosis (as shown by single positive for annexin V in the right lower quadrant in Figure 6a) as well as apoptotic cells in the process of undergoing secondary necrosis (cells in the right upper quadrant in figure 6b). In addition, the accumulation of collagen-producing cells was also reduced to nearly zero in all samples (Figure 6c-e). Due to the extremely low frequency of Pro-Col-Iα1^+ ^cells in these samples, further phenotyping could not be performed. These data indicate that apoptotic cell death responses promote collagen production in human monocytes and confirm the human relevance of our murine findings.

## Discussion

These studies provide new insight into the relationship of collagen-producing leucocytes and fibrotic lung disease. Specifically, they demonstrate that lung-targeted overexpression of TGF-β1 induces the intrapulmonary accumulation of a heterogeneous population of collagen-containing leucocytes, many of which express a cell surface phenotype characteristic of monocytes but appear to be distinct from alternatively activated macrophages. Furthermore, inhibition of cellular apoptosis results in a significant reduction in all of these populations and restores the CD45^+^Col-Iα^+ ^cell surface phenotype seen in wild-type mice. The human relevance of these findings is demonstrated by recapitulation of these results in the lungs and circulation of patients with two separate forms of fibrotic lung disease. Taken together, these data suggest that in the setting of apoptotic injury, monocytes adopt a reparative program characterized by enhanced production of collagen I.

The identity of the collagen producing leucocytes in our study is not entirely clear at this time but based on the robust expression of CD34 seen the cultured human cells, these cells are likely to be fibrocytes in intermediate state of differentiation. Fibrocytes were first described as blood-borne, fibroblast-like cells that appeared in exudative fluid at the earliest phases of wound repair [2]. They are considered to originate from CD14^+ ^myeloid cells and coexpress collagen I, CD45, and the progenitor marker CD34 though this latter marker is downregulated as these cells mature *in situ *[28]. CD34 is also lost on human fibrocytes during *in vitro *culture in the setting of TGF-β1 [12] suggesting that CD34 may be an early fibrocyte marker which is lost as the cell matures or is activated or that, as is seen in other settings [29,30], TGF-β1 exposure preferentially impedes the proliferation and survival of CD34^+ ^cells. Our findings support this paradigm and are consistent with prior reports in murine models of both asthma [28] and bleomycin induced lung fibrosis [12] showing that CD34^+ ^is rapidly lost upon entry into diseased tissue. Fibrocytes demonstrate a remarkably plastic phenotype, adopting the functional characteristics of both macrophages and fibroblasts in response to local cues. Thus, if the CD14^+ ^collagen expressing cells seen in our model are indeed fibrocytes, it is possible that their contributions to disease in this setting would include the fibrocyte functions typically attributed to macrophages such as cytokine and chemokine production [9], antigen presentation [31], inflammatory cell trafficking and activation [9] and promotion of angiogenesis [32], as well as the extracellular matrix (ECM)-producing and wound contractile properties typically attributed to activated myofibroblasts. Further studies investigating the role of collagen-producing leukocytes in lung fibrosis could be pursued in studies which live collagen-producing cells are isolated for *in vitro *studies and functional analysis. In addition, while it is assumed that these cells are producing collagen, it remains possible that the CD14^+ ^cells have engulfed collagen. This question highlights the need for the development of high affinity antibodies that detect the immature form of murine collagen.

It is also noteworthy that while our human findings recapitulated much of what was seen in the mouse, there were a number of differences. For example, CD45^+^Col-Iα1^+ ^cells in the TGF-β1-exposed lung were enriched for CD14 expression in the absence of CD34. In contrast, while the CD45^+^Pro-Col-Iα1^+ ^cells obtained from obtained from patients with lung disease also expressed high levels of CD14, many of these cells also expressed CD34. These data are could be related to the differences in tissue compartments studied (blood versus lung), the use of a transgenic TGF-β1 modeling system in the mouse, and/or unaccounted for clinical factors in the patients such as age and comorbidities. However, because we did not further phenotype the intrapulmonary CD45^+^Pro-Col-Iα1^+ ^cells from humans, comparison with the animal findings are limited at best. The mechanistic significance of CD34 on collagen-producing human leukocytes could perhaps best be examined in studies in which these cells are subdivided based on CD34 expression and then adoptively transferred into murine models of experimentally induced lung fibrosis.

In our studies, the *in vivo *inhibition of apoptosis in two different cell types, murine lung epithelia and human peripheral blood monocytes, significantly reduced the appearance CD45^+^Pro-Col-Iα1^+ ^cells, suggesting that this phenotypic change is a non-specific response to local cell death. Additional investigation will be required to determine why apoptosis is required for maximal accumulation of CD45^+^Col-Iα1^+ ^cells. Given the well documented effects of apoptotic bodies on monocyte biology [33,34], it is possible that the increased production of collagen by monocytes is a direct response to exposure to dead/dying cells as has been previously shown in elegant studies of cultured murine monocytes [33]. This hypothesis is further supported by data from studies demonstrating a reduction in CD45^+^Col-Iα1^+ ^cells upon exposure to the short pentraxin serum amyloid P, which modulates monocyte phenotypes in response to engulfment of apoptotic cells [35,36]. It is also possible that other cell populations such as lymphocytes or other monocyte derived cells respond to apoptosis by increasing the secretion of soluble mediators, such as semaphorin 7a [25], Stromal Derived Factor (SDF-1) [4] and Monocyte Chemotactic Protein-1 (MCP1) [37] that could promote the appearance of fibrocytes. In addition, caspase activation itself could induce monocyte abnormalities that lead to enhanced production of collagen as a form of immunosenesence. The documentation of elevated levels of CD45^+^Col-Iα1^+ ^cells in the senescence associated mouse [38], as well as in CD14^+ ^cells derived from aged but otherwise healthy humans [6], supports this latter hypothesis. Further work is needed to define the precise relationship between caspase activation, apoptosis, and the accumulation of CD45^+^Pro-Col-Iα^+ ^cells in the TGF-β1-exposed lung and in patients with pulmonary fibrosis.

Our studies also provide novel insight into the relationship between CD45^+^Col-Iα1^+ ^cells and CD206+ macrophages. We have previously shown that TGF-β1 induced lung fibrosis is dependent upon M2 macrophage accumulation [3]. In the current study we find that apoptosis is required for the appearance of CD45+Col-Iα cells but has a lesser effect on macrophages. Because CD206 is a robust marker of alternative activation [39], these data suggest that accumulation of M2 macrophages alone is insufficient for the development of TGF-β1-induced fibrosis and remodeling. When viewed in combination, these studies support a paradigm in which the profibrotic effects of TGF-β1 require both alternatively activated macrophages and collagen-producing leucocytes for maximal effect. The functional contributions of these populations will require further investigation.

## Conclusions

In summary, our studies demonstrate that local apoptotic responses potently stimulate the recruitment of collagen containing leucocytes in the TGF-β1-exposed murine lung. These CD45^+^Col-Iα1^+ ^cells exhibit significant phenotypic heterogeneity and appear in response to apoptotic cell death. These effects are seen in monocytes derived from patients with two separate forms of fibrotic lung disease, as well as in monocytes obtained from normal controls. These findings suggest that targeting apoptotic responses in an effort to attenuate collagen production by monocytes and the accumulation of fibrocytes may be beneficial in diseases of lung remodeling and aberrant repair.

## Materials and methods

### Transgenic mice

All mouse experiments were approved by the Yale School of Medicine Institutional Animal Care and Use Committee. The CC10-tTS-rtTA-TGF-β1 transgenic mice used in this study have been described [18]. These mice use the Clara cell 10-kDa protein (CC10) promoter to specifically express bioactive human TGF-β1 to the lung, and were backcrossed for > 10 generations onto a C57BL/6 background [18].

### Doxycycline administration

CC10-tTS-rtTA-TGF-β1 transgene positive (Tg^+^) or their wild-type littermate controls (transgene negative, Tg^-^) aged 8-10 weeks old were given doxycycline 0.5 mg/ml in their drinking water for up to 2 weeks.

### Lung inflammation

Mice were killed and bronchoalveolar lavage (BAL) performed as previously described [18]. Lung inflammation was assessed by assessing BAL samples as described previously [18].

### Collagen assessment

Total right lung collagen was measured using the Sircol Assay following manufacturer's protocol (Biocolour, Carrickfergus, Ireland).

### Flow cytometry

Lung samples were digested for flow cytometric identification of CD45^+ ^Col-Iα1^+ ^cells as previously described [3]. Total viable cells were quantified using Trypan blue staining. Collagen-producing leukocytes were detected using CD45 surface staining (BD Pharmingen, San Jose, CA) and intracellular staining for Col-Iα1 (Millipore, Billerica, MI). Flow cytometric analysis of CD45^+^Col-Iα1^+ ^cells was performed by identifying live cells based on forward and side scatter characteristics, gating on the CD45^+ ^cells, and then gating on the Col-Iα1^+ ^cells within this population. Cells were then further subgated based on their expression of CD34 and/or CD14. Percentages of live cells coexpressing these markers were multiplied by total viable cell count of digested sample to determine the absolute number of collagen containing leucocytes.

### TUNEL

TUNEL was performed as previously described [3].

### Caspase activation

Detection of caspase cleavage and activation using immunohistochemistry was performed as previously described [18].

### Annexin V

Flow cytometric assessment of annexin V externalization was performed via flow cytometry as previously described [18].

### Histological analysis

Formalin-fixed and paraffin-embedded lung sections were stained with hematoxylin and eosin to assess gross morphology or Mallory's trichrome stains to visualize collagen deposition.

### Human cell isolation and culture

All studies were performed with HIC approval and written informed consent at Yale University School of Medicine. Individuals with no known medical conditions who self-identified as healthy were included as controls. Patients with SSc-ILD or amyopathic antisynthetase syndrome according to American College of Rheumatology (ACR) criteria or IPF according to current European Respiratory Society (ERS)/American Thoracic Society (ATS) criteria [40] were recruited as the study group. Exclusion criteria included concurrent diagnosis of malignancy, pregnancy, the presence of known secondary lung disease such as pulmonary hypertension or chronic airway obstruction or inability (due to psychiatric or language limitations) to provide informed consent. A total of 30 ml of peripheral blood was drawn, peripheral blood mononuclear cells (PBMCs) isolated via density gradient centrifugation and CD14+ monocytes were enriched as previously described by our group [26]. Cells were cultured in 96 well plates in the presence or absence of 100 μM Z-VAD/fmk (EMD Biochemical, Los Angeles, CA). After 10 days of culture cells were assessed qualitatively for fibrocytes based on spindle-shaped morphology. Cells were then harvested and assessed for CD45^+^Col-Iα1^+ ^phenotype by fluorescence-activated cell sorting (FACS) as previously described [6].

### Flow cytometry of human cells

Antibodies against human CD45, CD34, CD14, and appropriate isotype controls were obtained from BD Pharmingen. Flow cytometry and cell sorting was performed using a BD FACSCalibur. Data were analyzed using Flow Jo v 7.5 software (TreeStar, Inc, Ashland, OR). For all analyses, isotype control staining was subtracted from true antibody staining to determine the percentage of positive cells.

### Statistics

Gaussian distribution of data was determined using the D'Agostino and Pearson omnibus normality test. Normally distributed data are expressed as means ± SEM and assessed for significance by Student's *t *test or analysis of variance (ANOVA) as appropriate. Data that were not normally distributed were assessed for significance using the Mann-Whitney U test where appropriate.

## Competing interests

ELH received grant funding from Promedior and Medimmune, though neither of these entities supported the studies performed in this manuscript.

## Authors' contributions

XP performed the human and animal studies. SKM performed flow cytometry on human samples and assisted in preparation of this manuscript. LM contributed to study design, data interpretation, and manuscript preparation. TR performed human studies. RR performed human studies. QC and YG performed animal studies. MG performed statistical analyses on the data. JE and RB assisted in the drafting of this manuscript. ELH participated in study design, data interpretation, and manuscript preparation. All authors read and approved the final manuscript.
